# Supplementary material for: Unravelling reference bias in ancient DNA datasets
Source: Bioinformatics. 2024 Jul 3;40(7):btae436. doi: 10.1093/bioinformatics/btae436 (PMC11254355; doi:10.1093/bioinformatics/btae436)
Supplement: btae436_Supplementary_Data [file btae436_supplementary_data.zip › S1-S4_data_plots.zip!/S1-S4_data/elephant_sim_plots.zip!/elephant_sim_plots/bwamem_elephant_div1_10end_by_MQ.pdf]

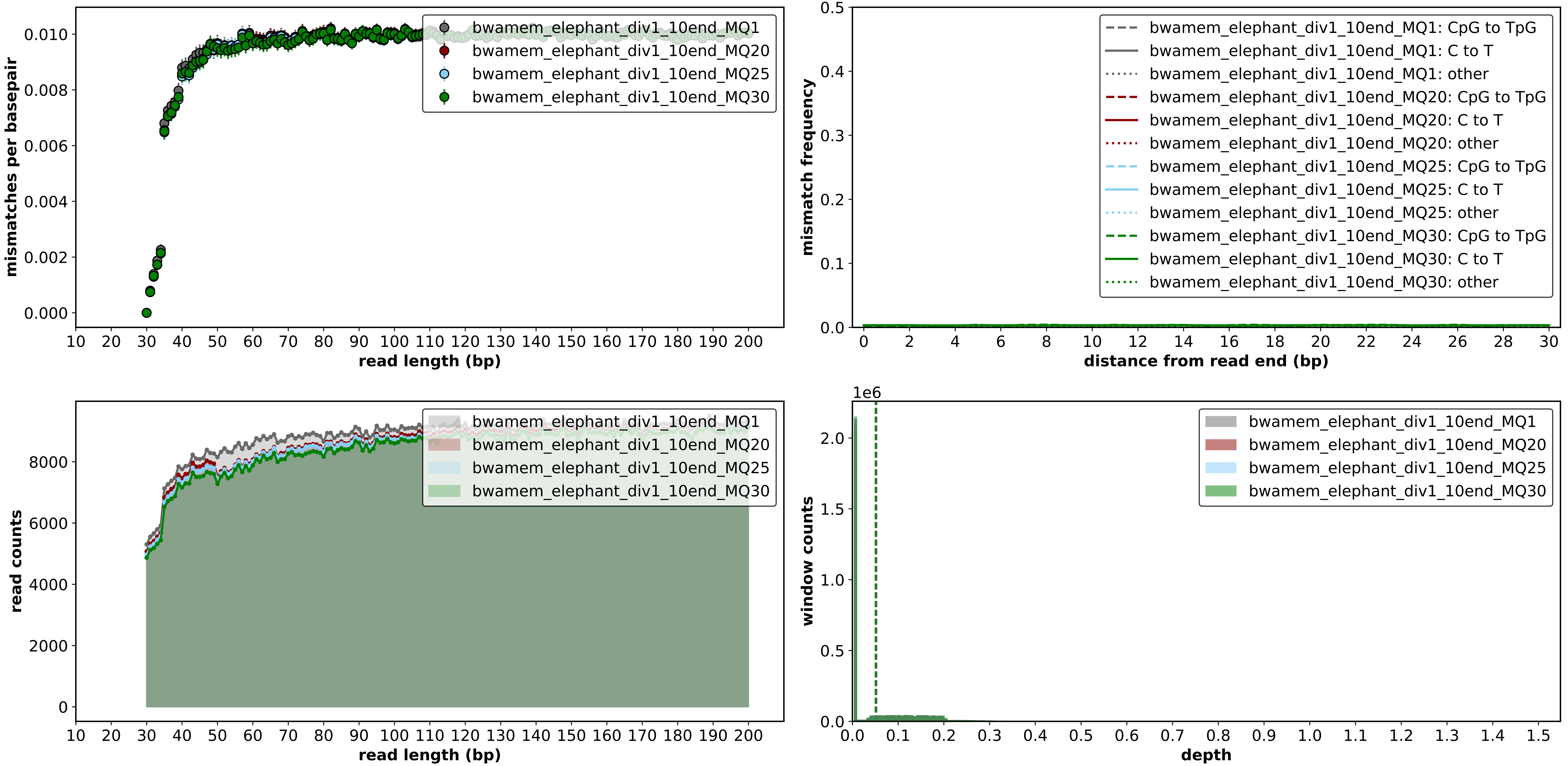
mismatches per basepair
0.000
0.002
0.004
0.006
0.008
0.010
read length (bp)
10 20 30 40 50 60 70 80 90 100 110 120 130 140 150 160 170 180 190 200
bwamem_elephant_div1_10end_MQ1
bwamem_elephant_div1_10end_MQ20
bwamem_elephant_div1_10end_MQ25
bwamem_elephant_div1_10end_MQ30
mismatch frequency
0.0
0.1
0.2
0.3
0.4
0.5
distance from read end (bp)
0 2 4 6 8 10 12 14 16 18 20 22 24 26 28 30
bwamem_elephant_div1_10end_MQ1: CpG to TpG
bwamem_elephant_div1_10end_MQ1: C to T
bwamem_elephant_div1_10end_MQ1: other
bwamem_elephant_div1_10end_MQ20: CpG to TpG
bwamem_elephant_div1_10end_MQ20: C to T
bwamem_elephant_div1_10end_MQ20: other
bwamem_elephant_div1_10end_MQ25: CpG to TpG
bwamem_elephant_div1_10end_MQ25: C to T
bwamem_elephant_div1_10end_MQ25: other
bwamem_elephant_div1_10end_MQ30: CpG to TpG
bwamem_elephant_div1_10end_MQ30: C to T
bwamem_elephant_div1_10end_MQ30: other
read counts
0
2000
4000
6000
8000
read length (bp)
10 20 30 40 50 60 70 80 90 100 110 120 130 140 150 160 170 180 190 200
bwamem_elephant_div1_10end_MQ1
bwamem_elephant_div1_10end_MQ20
bwamem_elephant_div1_10end_MQ25
bwamem_elephant_div1_10end_MQ30
1e6
window counts
0.0
0.5
1.0
1.5
2.0
depth
0.0 0.1 0.2 0.3 0.4 0.5 0.6 0.7 0.8 0.9 1.0 1.1 1.2 1.3 1.4 1.5
bwamem_elephant_div1_10end_MQ1
bwamem_elephant_div1_10end_MQ20
bwamem_elephant_div1_10end_MQ25
bwamem_elephant_div1_10end_MQ30
